# Prediction of bone metastasis of prostate cancer based on intratumoral and peritumoral radiomics of MRI T2WI combined with ADC images

**DOI:** 10.3389/fonc.2025.1555315

**Published:** 2025-03-10

**Authors:** Shiqian Lin, Pingping He, Ruixiong You

**Affiliations:** ^1^ Department of Radiology, First Affiliated Hospital of Fujian Medical University, Fuzhou, China; ^2^ Department of Radiology, Clinical Oncology School of Fujian Medical University, Fujian Cancer Hospital, Fuzhou, China

**Keywords:** magnetic resonance imaging, T2WI, ADC, prostate cancer, bone metastasis, radiomics

## Abstract

**Objective:**

To investigate the value of intratumoral and peritumoral MRI radiomic models in predicting bone metastasis of prostate cancer patients using T2WI combined with ADC images.

**Materials and method:**

A total of 144 patients with prostate cancer who underwent preoperative MRI (T2WI and DWI) were retrospectively included. All patients were categorized into two groups based on the presence of bone metastasis. The radiomics features were calculatd for the entire tumor and 3mm-peritumoral components on pre-processed T2WI combined with ADC images. The radiomics models based on intratumoral features, peritumoral features as well as their merged features were respectively constructed. The independent risk factors of bone metastasis of prostate cancer were used to constructed clinical prediction model. The performance of the clincal model, radiomics models and clinic-imaging combined models was evaluated by the receiver operating characteristic curve and compared with the bootstrap methods. T-test was used to compare the evaluation indicators of different prediction models.

**Results:**

The clinic-imaging combined model had the best predictive efficacy among all models. The area under the curve (AUC) of the clinic-imaging combined model for predicting bone metastasis of prostate cancer in the training dataset and test dataset were 0.937 and 0.893, respectively. The accuracy, sensitivity and specificity of this model in predicting bone metastasis of prostate cancer in the training dataset were 84.2%, 91.2% and 80.6%, respectively; the accuracy, sensitivity and specificity of the testing dataset were 76.7%, 73.3% and 78.6%, respectively.

**Conclusions:**

T2WI and ADC intratumoral and peritumoral radiomic models can be used to noninvasively predict the primary diagnosis of PCa BM, and peritumoral radiomic model can add independent predictive value. And the clinic-imaging combined model has the better predictive value.

## Introduction

Prostate cancer (PCa) is the world’s second most frequent cancer and the fifth leading cause of cancer death among males (1). Bone is the most common metastatic site of PCa, accounting for more than 80% of all (2, 3). Patients with bone metastasis (BM) in PCa tend to have worse prognosis and higher mortality than the patients without BM (4, 5). Whether bone metastasis occurs affects the final clinical treatment plan of patients. However, the vast majority of patients with BM in PCa have no obvious symptoms in the early stages, so they often present with BM at the initial visit (6). Therefore, early and accurate identification of the PCa BM is useful for individualizing strategies for treatment and improving patients’ survival prognosis.

The whole-body skeletal imaging using ^99^m Technetium Methylene Diphosphonate (^99^mTc-MDP) is currently the preferred method for PCa BM screening. Positron Emission Tomography-Computed Tomography (PET-CT) can provide more detailed images with high diagnostic accuracy (7). CT could also be used to evaluate BM. However, these examinations have lots of disadvantages including radiation exposure and low specificity which limits clinical application (8). Traditional MRI has high sensitivity and specificity in diagnosing BM. While the evaluation using traditional MRI obtained few quantitative features and relatively poor repeatability (9, 10). Therefore, more objective and effective methods need to be explored to predict the occurrence of PCa BM.

Previous studies have confirmed that MRI radiomics has good effects in the diagnosis, efficacy evaluation, recurrence prediction, and other aspects of PCa patients (11–14). Radiomics is the process method of extracting a large amount of information from medical images through converting digital images into quantitative high-dimensional data, which could characterize histologic or pathophysiologic information. MRI radiomics was also used for prediction of PCa BM, Wang et al. found that multiparametric MRI-based texture features were significant predictor for BM in PCa (AUC=0.898) (15).

However, most of the PCa radiomics studies mainly focus on the extraction and related analysis of radiomics features of the primary tumor lesion other than the surrounding area of the lesion. The intratumoral radiomics features may not characterize the information of tumors fully. Ding et al. found that the prediction model of lymph node metastasis of breast cancer combined with intratumoral and peritumoral radiomic features is more accurate than the single intratumoral radiomic model (16). Zhang et al. found that peritumoral radiomic model outperformed both only intratumoral or peritumoral combined with intratumoral radiomic model in predicting recurrence-free survival in hepatocellular carcinoma (17). MRI peritumoral radiomic features may provide additional predictive or diagnostic value for tumors, but the study using peritumoral radiomics to predict PCa BM remains unclear.

Thus, our study established and analyzed the intratumoral and peritumoral MRI radiomics models in PCa patients. T2WI and DWI are the most commonly recommended sequences in PCa MRI (18). ADC is the main evaluation parameter of DWI, and numbers of previous studies have converted the DWI images to ADC maps for further research (9, 19), so we chose T2WI and ADC images for further study. We aimed to investigate the value of radiomic models in predicting PCa BM and determine whether peritumoral radiomics can provide additional prediction value.

## Materials and methods

### Patients

We retrospectively enrolled PCa patients who underwent prostate MRI examination and prostate biopsy or radical resection surgery at the First Affiliated Hospital of Fujian Medical University, whose detailed pathological results were obtained after biopsy or surgery from July 2017 to December 2023.

The inclusion criteria were as follows: (1) Not undergoing biopsy or surgery prior to MRI examination; (2) Complete MRI examination images(including T2WI, DWI and ADC images) and clinical laboratory data; (3) Preoperative PET-CT or whole-body skeletal imaging examination, which is performed within 4 weeks after the MRI examination.

The exclusion criteria were as follows: (1) History of radiotherapy, chemotherapy, hormone therapy, or targeted therapy prior to MRI examination; (2) Due to the presence of other primary malignant tumors, the origin of BM cannot be determined; (3) Combined with a history of other bone injuries and bone metabolism disorders; (4) Poor image quality and artifacts make it impossible to evaluate; (5) PCa lesions with a length diameter of less than 1cm. We enrolled 144 patients in this study finally. We collected clinical and pathological data of patients, including age, the Serum Total Prostate Specific Antigen (tPSA), Gleason Score (GS) and Clinical Tumor-staging x (cTx).

### Image data acquisition

All patients underwent preoperative prostate MR imaging on 3.0 MR scanner (Siemens Skyra, Siemens Magnetom Verio, Siemens Prisma, Philips Ingenia) with phased array coils on the body. The sequences included transverse T2WI and DWI. The corresponding ADC maps are automatically fitted and generated by the MRI post-processing system. The sequences parameters for the different machines are shown in **Supplementary Table S1**.

### Image data preprocessing

All MRI images underwent a standard image preprocessing protocol. N4 bias field correction was applied to all images before further processing to reduce intensity inhomogeneity using Simple ITK. We used the REST plus software (version 1.25, http://restfmri.net/forum/RESTplus) based on the MATLAB R2022a platform to perform image resampling to minimize the effect of differences in image spatial resolution on the histological features of the images, all MRI images and ROIs were resampled to 1 mm × 1 mm × 1 mm. Additionally, ANTs (Advanced Normalization Tools, http://stnava.github.io/ANTs) was used to align ADC sequences to the T2WI sequences.

### Tumor segmentation

The volumes of interest (VOIs) of lesions were segmented using 3D Slicer software (version 5.4.0, https://www.slicer.org/). These VOIs were manually annotated layer by layer along the tumor margins by a radiologist (radiologist A) with 3 years’ diagnostic experience in diagnosis of genitourinary imaging. T2WI is widely used for prostate segmentation because it shows more detailed prostate anatomy than other MRI sequences. The aligned prostate segmentation can be applied to other image sequences (9). The specific delineated method was as follows: (1) Intratumoral radiomic model: we manually outlined the VOI of the lesion on the pre-processed MRI transaxial T2WI images; (2) Peritumoral radiomic model: on the basis of the intratumoral VOI, the peritumoral region was automatically expanded in 3D space by using the “Hollow” algorithm in the 3D Slicer software, starting from the tumor boundary, and the range of the expansion was 3 mm. At the same time, the VOI of the tumor drawn earlier was transformed into a hollow volume. When the expanded VOI exceeded the prostate parenchyma, the exceeded portion was manually erased.

### Feature extraction

The Pyradiomics module in the opensource software FeAture Explore (FAE, version 0.5.8) based on Python (version 3.7.6) was used to perform quantitative radiomics feature extraction on the outlined VOI images. All images were pre-processed before feature extraction. The pre-processing steps include normalization, Mask re-segmentation and Discretization.

A total of 851 features were extracted from each VOI of each sequence including a set of original image features (14 morphological, 18 first-order statistical and 75 texture features) and 8 sets of wavelet transformed image features (18 first-order statistical and 75 texture features). The T2WI and ADC imaging features were obtained using the above methods. Finally, 1702 radiomics features were obtained for each of the tumor lesion and peritumoral area. The specific radiomic features extracted for this study are shown in **Supplementary Table S2**.

### Data preprocessing and feature selection

This study applies the Interclass Correlation Coefficient (ICC) for preliminary screening of the extracted features to minimize the impact of VOI segmentation bias on the construction of radiomics models. Thirty lesions were randomly chosen for repetitive VOI segmentation, which was performed by two radiologists to explore the intra-observer and inter-observer stability of features. The radiomics features with both ICC values > 0.75 were retained for further study.

All patients were randomly grouped into the training and testing datasets in a ratio of approximately 7:3. The Mean value method was applied to all feature matrices for feature normalization. We used the Pearson correlation coefficient (PCC) to reduce the dimensions of the row space of the feature matrix. Then, we used Recursive feature elimination (RFE) to select the most relevant feature subset. The Synthetic minority oversampling technique (SMOTE) was used to improve learning via imbalanced datasets to reduce the influence of data imbalance.

### Development of prediction model

For radiomics model construction, we selected five classifiers, namely, Gaussian process (GP), Logistic regression (LR), Linear discriminant analysis (LDA), Random forest (RF), and Support vector machines (SVM) to fit the data. Ultimately, five-fold cross-validation was applied to select the final features from the most relevant feature subset. The number of final selected features was set according to the performance of cross-validation in the balanced training cohort.

The model with the highest Area under the Curve (AUC) in the validation set was selected as the optimal model to establish the radiomics model including intratumoral model and peritumoral model. The Prediction Value (Pred) of BM in each case was automatically calculated from the final model. The radiomic features extracted from the intratumoral and peritumoral VOIs were combined to construct a combinatorial radiomic model, which is denoted as “Merged model”. The above procedures were realized on FeAture Explore (FAE, version 0.5.8) software based on Python (version 3.7.6).

**Figure 1:**
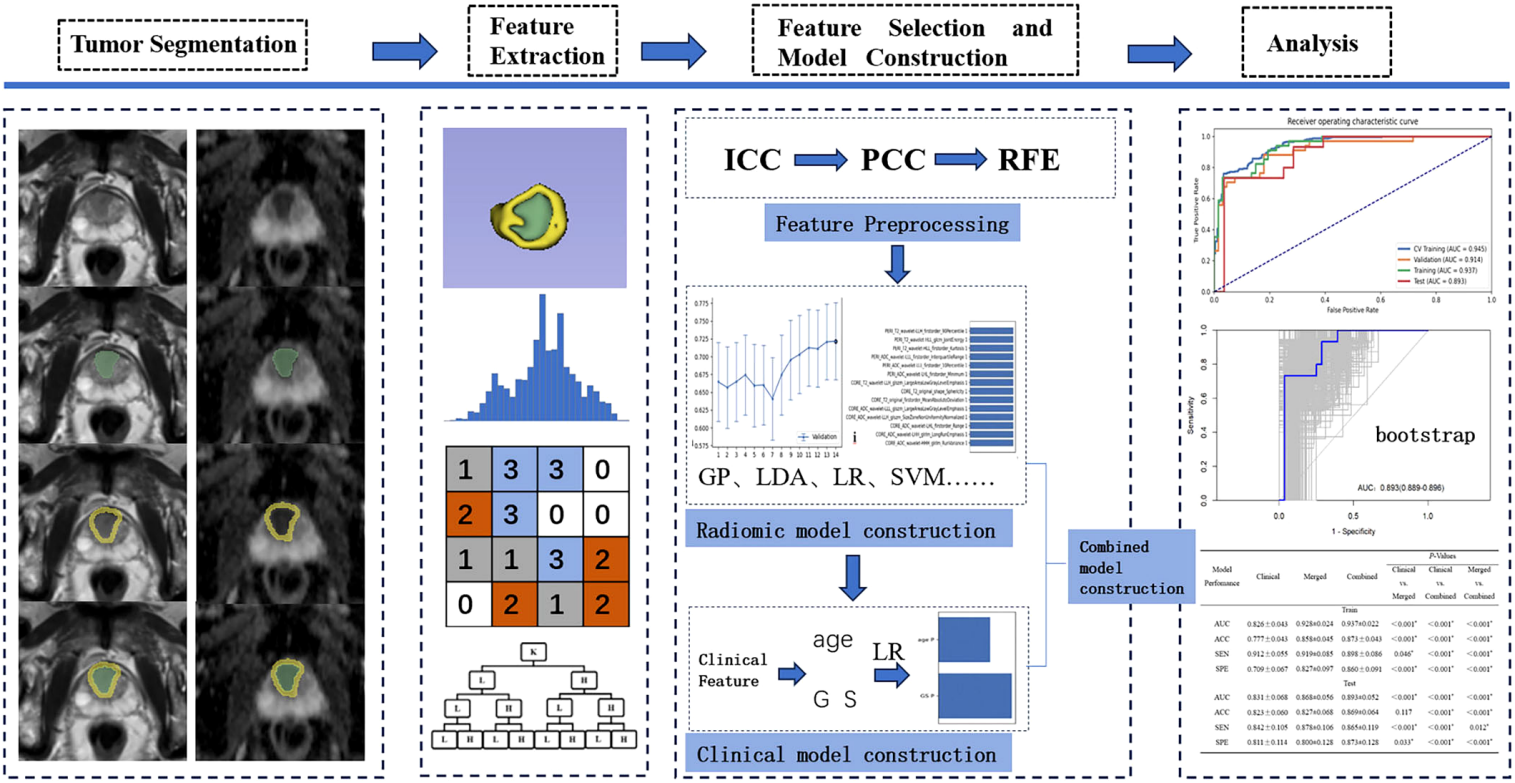
The workflow chart of this study.

**Figure 2:**
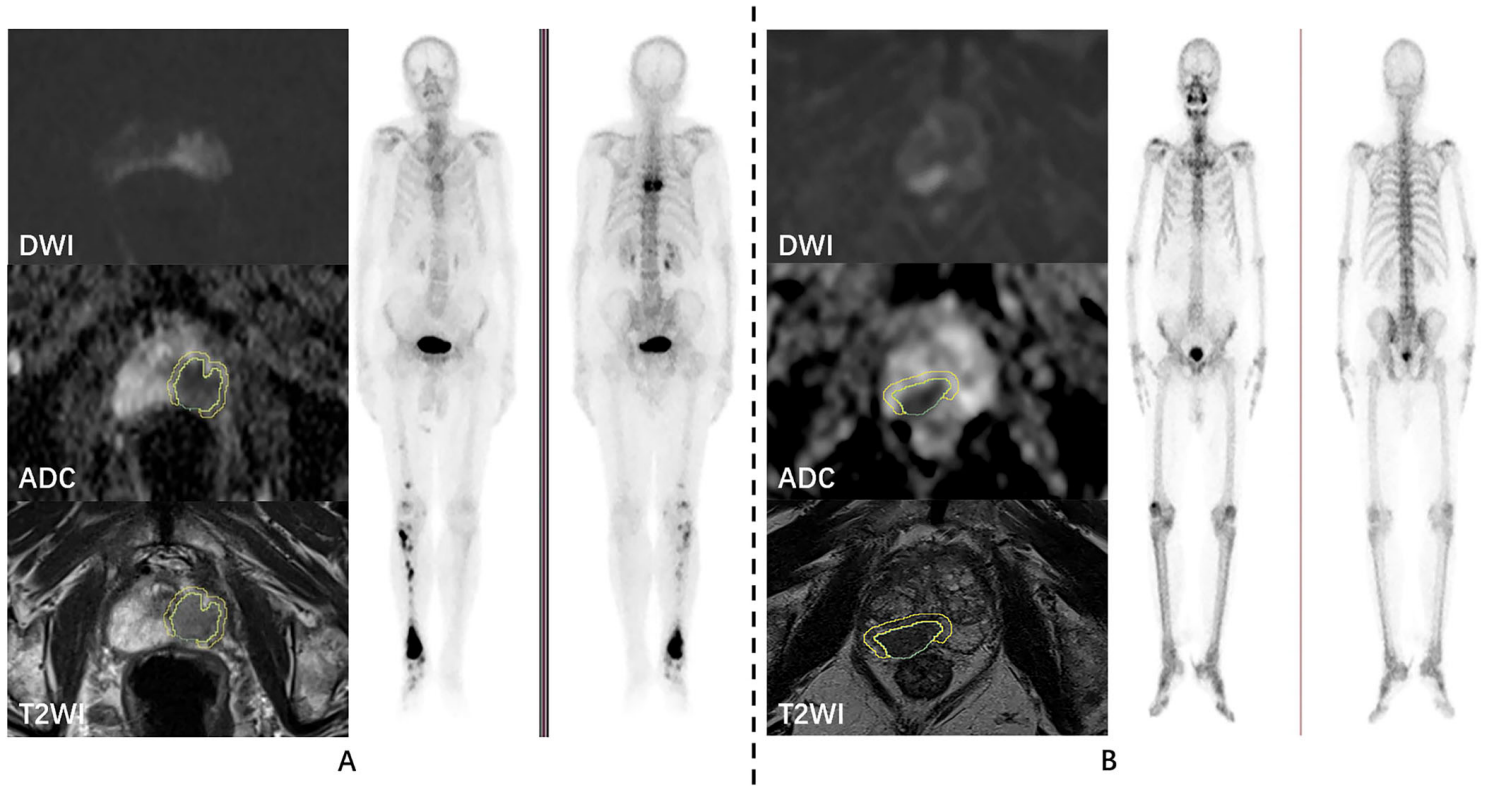
Examples of prostate cancer patients. DWI, ADC, T2WI, and whole-body skeletal imaging images of prostate cancer patients with bone metastasis **(A)** and without bone metastasis **(B)**, respectively.

The clinical variables in the training cohort that exhibited statistically significant differences between the BM and non-BM groups were screened for clinical model construction using the LR classifier. The Pred of the radiomic model with the highest AUC in the cross-validation set and clinical model was selected for clinic-imaging combined model construction. **Figure 1** shows the workflow of this study. **Figure 2** shows two examples of prostate cancer patients.

### Statistical analysis

Statistical analysis was carried out using R software (version 4.3.2, http://www.Rprogject.org) and SPSS software (version 27.0.1.0). Continuous variables were compared by using the Student’s t-test or the Mann−Whitney U test.Categorical variables were compared using the Chi-square test or Fisher’s exact test. Independent risk factors associated with bone metastasis were screened using univariate and multivariable logistic regression analysis. The performance of the models was evaluated by the receiver operating characteristic curve, and the area under the curve (AUC), accuracy (ACC), sensibility (SEN), and specificity (SPE) were calculated. The precise-recall (PR) curve was used to valuated the model performance additionally.

To further compare the performances of the models, we performed bootstrap random resampling 1000 times on each dataset. Based on the evaluated variation for each model performance, we used the paired t-test to identify the significant differences of AUC,ACC,SEN and SPE between the predictive models with Benjamini-Hochberg correction for the multiple comparisons (20). All *P*-values are two-sided test results, and *P* < 0.05 was considered indicative of statistical significance.

## Results

### Patients’ characteristics

According to the inclusion and exclusion criteria, a total of 144 PCa patients were finally included in the analysis, including 49 patients in the BM group and 95 patients in the non-BM group.

The distribution of patients’ age, tPSA, GS, and cTx between the BM and non-BM groups was statistically different (*P* < 0.05). The distribution of clinical and pathologic data is shown in **Table 1**.

**Table 1:** Patients’ clinical and pathological information.

Features	BM (n = 49)	Non-BM (n = 95)	*P-value*
age (year)	73.71 ± 7.54	69.38 ± 8.20	0.003^*^
tPSA (ng/ml)	84.18 [31.60,100.00]	19.00[11.80,39.41]	< 0.001^*^
GS			< 0.001^*^
GS < 7	1 (2.04%)	9 (9.47%)	
GS = 7	5 (10.20%)	60 (63.16%)	
GS > 7	43 (87.76%)	26 (27.37%)	
cTx			< 0.001^*^
T2a	3 (6.12%)	12 (12.63%)	
T2b	2 (4.08%)	10 (10.53%)	
T2c	5 (10.20%)	35 (36.84%)	
T3a	13 (26.53%)	29 (30.53%)	
T3b	21 (42.86%)	9 (9.47%)	
T4	5 (10.20%)	0 (0.00%)	

BM, bone metastasis; tPSA, the Serum Total Prostate Specific Antigen; GS, Gleason Score; cTx, clinical tumor-staging x. *P < 0.05, represents significant difference between groups.

There were 101 patients in the training dataset (BM = 34, non-BM = 67) and 43 patients in the testing dataset (BM = 15, non-BM = 28). In the training dataset, the differences in age, tPSA, GS, and cTx of patients were statistically significant (*P* < 0.05). There was no significant difference in the distribution of baseline information between the training and testing datasets (*P* > 0.05). The distribution and comparison of baseline information between the training and testing datasets are shown in **Table 2**.

**Table 2:** Clinical and pathological information between patients with BM and with non-BM in the training dataset and test dataset.

Feature	the training dataset (n=101)			the test dataset (n=43)			*P^c^* value
	BM (n=34)	Non-BM (n=67)	*P^a^* value	BM (n=15)	Non-BM (n=28)	*P^b^* value	
Age (year)	74.44 ± 7.77	69.81 ± 7.64	0.006^*^	72.07 ± 6.71	68.36 ± 9.32	0.190	0.256
tPSA (ng/ml)	68.00 [21.70,100.00]	18.00 [10.20, 40.20]	< 0.001^*^	84.18 [36.00,100.00]	20.50 [14.90,33.00]	0.003^*^	0.508
GS			< 0.001^*^			< 0.001^*^	0.222
GS < 7	1 (2.94%)	8 (11.94%)		0 (0.00%)	1 (3.57%)		
GS = 7	3 (8.240%)	39 (58.21%)		2 (13.33%)	21 (75.00%)		
GS > 7	30 (88.24%)	20 (29.85%)		13 (86.67%)	6 (21.43%%)		
cTx			< 0.001^*^			0.009^*^	0.656
T2a	3 (8.82%)	7 (10.49%)		0 (0.00%)	5 (17.86%)		
T2b	2 (5.88%)	8 (11.94%)		0 (0.00%)	2 (7.14%)		
T2c	3 (8.82%)	25 (37.31%)		2 (13.33%)	10 (35.71%)		
T3a	9 (26.47%)	21 (31.34%)		4 (26.67)	8 (28.57%)		
T3b	15 (44.18%)	6 (8.96%)		6 (40.00%)	3 (10.71%)		
T4	2 (5.88%)	0 (0.00%)		3 (20.00%)	0 (0.00%)		

BM, bone metastasis; tPSA, the Serum Total Prostate Specific Antigen; GS, Gleason Score; cTx, clinical tumor-staging x; *P^a^* represents the statistically significant level of the difference between the two groups in the training set, *P^b^* represents the statistically significant level of the difference between the two groups in the test set, and *P^c^* represents the statistically significant level of the feature distribution difference between the training group and the test group. *P < 0.05, represents significant difference between groups.

The results of univariate logistic regression analysis of the training set showed that age, tPSA, GS and cTx were all associated risk factors for BM in PCa (*P* < 0.05); the results of multivariable logistic regression analysis of the training set showed that age and GS were independent risk factors for the development of BM in PCa patients (*P* < 0.05), as shown in **Table 3**.

**Table 3:** Univariate and multivariable logistic regression analysis of patients’ clinical and pathological features of the training dataset.

	Univariate logistic regression analysis		Multivariable logistic regression analysis	
	*P*	*OR (95%CI)*	*P*	*OR (95%CI)*
age	0.008^*^	1.084 (1.021-1.151)	0.040^*^	1.080 (1.003-1.162)
tPSA	0.030^*^	1.005 (1.000-1.010)	0.158	1.003 (0.999-1.007)
GS	< 0.001^*^	10.825 (3.774-31.047)	< 0.001^*^	6.513 (2.179-19.465)
cTx	0.001^*^	2.012 (1.326-3.052)	0.290	1.286 (0.807-2.050)

BM, bone metastasis; tPSA, the Serum Total Prostate Specific Antigen; GS, Gleason Score; cTx, clinical tumor-staging x; *OR*, Odds Ratio, *CI*, Confidence Interval. *P < 0.05, represents significant difference between groups.

### Radiomic models constructing results

After the ICC analysis, 1414 intratumoral features and 901 peritumoral radiomic features with favorable reproducibility were retained, respectively, for subsequent analysis.

After dimensionality reduction and feature selection, LDA (AUC = 0.599 in the cross-validation cohort) were identified as the optimal classifier used for peritumoral radiomic model constructions, which incorporated 7 features. Intratumoral and merged radiomic models’ selection using the same methods described above. Intratumoral radiomics models based on SVM (AUC = 0.694 in the cross-validation cohort) were identified as the optimal classifier incorporating 3 features. The highest efficacy (AUC = 0.722) was found in the cross-validation set of the GP classifier-based merged radiomic model, which incorporated a total of 14 features.

### Clinical models constructing results

Independent risk factors from the training dataset including age and GS were incorporated into the LR classifier for clinical model construction. The AUC of the clinical model cross-validation set was higher when 2 features were included (AUC = 0.802).

### Clinic-radiomic combined model constructing results

**Figure 3:**
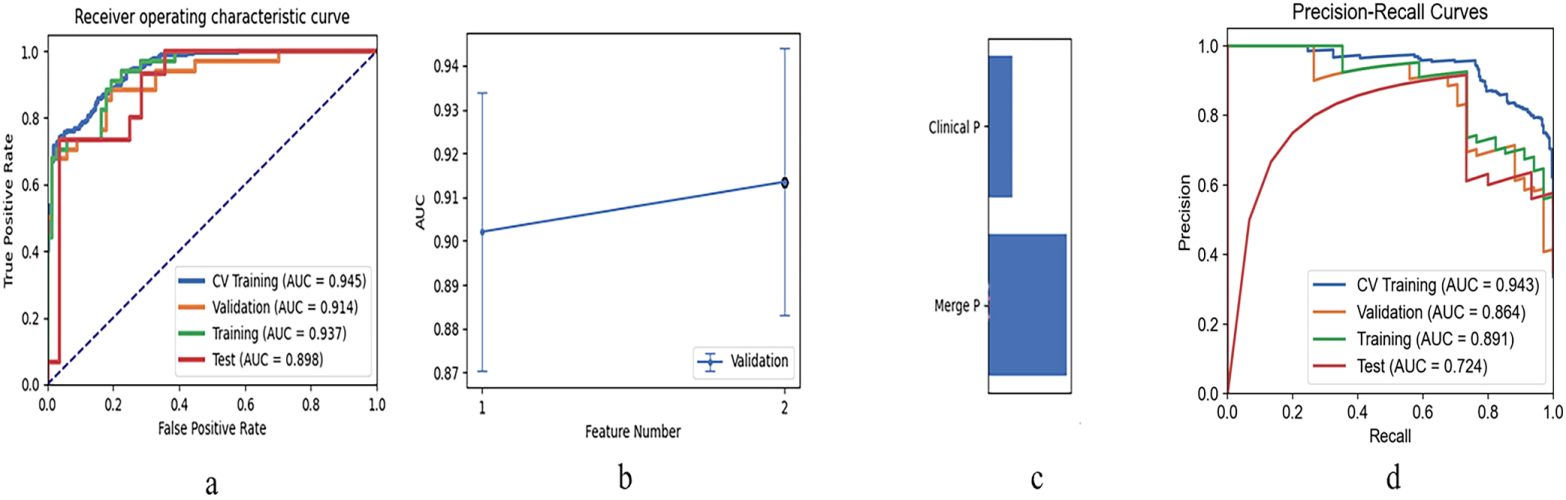
Performance of the clinic-radiomic combined model. **(a)** Receiver operating characteristic curves of the combined model using different datasets; **(b)** The AUC value of cross-validation dataset reached the highest value when this model contains 2 features; **(c)** Names and coefficients of the features in combined model; **(d)** Precision-Recall curves of the combined model using different datasets.

Among the above three radiomic models, the cross-validation set of the merged radiomic model had the highest efficacy (AUC = 0.722). the Pred of the merged model was combined with the Pred of the clinical model for the construction of the clinic-radiomic combined model. The model incorporating the predictions of both models had the higher AUC in the cross-validation set, with AUC = 0.937, ACC = 0.842, SEN = 0.912, SPE = 0.806, 95% CI [0.893-0.981] in the training dataset and AUC = 0.893, ACC = 0.767, SEN = 0.733, SPE = 0.786, 95%CI[0.793-0.993] in the testing dataset. The ROC curves of the clinic-radiomic combined model, the performance of different feature models in the validation set, and the feature contribution are shown in the **Figure 3**. The PR curves of the clinic-radiomic combined model are shown in the **Figure 3d**. The predictive performance of the peritumoral model, intratumoral model, merged model, clinical model and clinic-radiomic combined model are shown in **Table 4**.

**Table 4:** The predictive performance of the peritumoral model, intratumoral model, merged model, clinical model, and combined model.

Model type	Dataset	AUC	95%CI	Cutoff	ACC	SEN	SPE	AUPRC
Peritumoral Radiomic	Training	0.848	0.766-0.930	0.443	0.772	0.882	0.716	0.737
	Testing	0.788	0.653-0.923	0.443	0.721	0.533	0.821	0.637
Intratumoral Radiomic	Training	0.784	0.685-0.884	0.633	0.822	0.588	0.940	0.721
	Testing	0.850	0.738-0.962	0.633	0.721	0.400	0.893	0.714
Merged Radiomic	Training	0.928	0.880-0.975	0.485	0.852	0.853	0.851	0.866
	Testing	0.867	0.758-0.976	0.485	0.791	0.733	0.821	0.724
Clinical	Training	0.826	0.742-0.870	0.312	0.762	0.912	0.687	0.651
	Testing	0.833	0.702-0.964	0.312	0.791	0.867	0.750	0.671
Clinic-Imaging Combined	Training	0.937	0.893-0.981	0.390	0.842	0.912	0.806	0.891
	Testing	0.893	0.793-0.993	0.390	0.767	0.733	0.786	0.724

AUC, Area Under the Receiver Operating Characteristic Curve; CI, Confidence Interval; ACC, Accuracy; SEN, Sensibility; SPE, Specificity; AUPRC, Area Under the Precision-Recall Curve.

### Comparison of predictive efficacy of models

The performance of the intratumoral radiomic model, merged radiomic model, the clinical model and the combined model after bootstrap as well as the comparison of the statistical results between different models are shown in **Table 5**. Both in the training set and the testing dataset, the AUC, ACC, SEN, and SPE values of merged radiomic model were better than those of the intratumoral radiomic model (*P* < 0.05). The combined model was better than the merged radiomic model and the clinical model (*P* < 0.05). The differences of the predicted values of the combined model and those of the different models were statistically significant. In the testing dataset, the differences between the predicted values of the combined model and the different models were statistically significant (*P* < 0.05).

**Table 5:** Statistical comparisons between developed predictive models accepting bootstrap.

Model Performance	Intratumoral Radiomic model	Clinical model	Merged Radiomic model	Combined model	*P-value*			
					Merged vs. Intratumoral	Clinical vs. Merged	Clinical vs. Combined	Merged vs. Combined
the training dataset								
AUC	0.783 ± 0.051	0.826 ± 0.043	0.928 ± 0.024	0.937 ± 0.022	< 0.001*	<0.001^*^	< 0.001^*^	< 0.001^*^
ACC	0.813 ± 0.055	0.777 ± 0.043	0.858 ± 0.045	0.873 ± 0.043	< 0.001*	<0.001^*^	< 0.001^*^	< 0.001^*^
SEN	0.637 ± 0.114	0.912 ± 0.055	0.919 ± 0.085	0.898 ± 0.086	< 0.001*	0.046^*^	< 0.001^*^	< 0.001^*^
SPE	0.902 ± 0.108	0.709 ± 0.067	0.827 ± 0.097	0.860 ± 0.091	< 0.001*	<0.001^*^	< 0.001^*^	< 0.001^*^
the testing dataset								
AUC	0.818 ± 0.071	0.831 ± 0.068	0.868 ± 0.056	0.893 ± 0.052	< 0.001*	<0.001^*^	< 0.001^*^	< 0.001^*^
ACC	0.774 ± 0.073	0.823 ± 0.060	0.827 ± 0.068	0.869 ± 0.064	< 0.001*	0.117	< 0.001^*^	< 0.001^*^
SEN	0.925 ± 0.111	0.842 ± 0.105	0.878 ± 0.106	0.865 ± 0.119	< 0.001*	<0.001^*^	< 0.001^*^	0.012^*^
SPE	0.693 ± 0.136	0.811 ± 0.114	0.800 ± 0.128	0.873 ± 0.128	< 0.001*	0.033^*^	< 0.001^*^	< 0.001^*^

AUC, Area Under the Receiver Operating Characteristic Curve; CI, Confidence Interval; ACC, Accuracy; SEN, Sensibility; SPE, Specificity. ^*^Significant difference is identified based on the t-test; *P*-Values was corrected by Benjamini-Hochberg method. ^*^ *P* < 0.05, represents significant difference between groups.

## Discussion

In this study, we retrospectively analyzed the diagnostic efficacy of intratumoral and peritumoral MRI radiomic models based on T2WI and ADC images combined with some clinical data (including age and GS) to predict BM in PCa patients. The results showed that the intratumoral radiomic model was superior to the peritumoral radiomic model; the merged radiomic model improved diagnostic efficacy, and the merged radiomic model was superior to the clinical model. Combining clinical data and radiomic features could further improve the predictive efficacy of the model.

Numerous studies have shown that the tPSA level, GS and cTx can be used as predictors of whether patients need to be tested for PCa BM, and they are generally considered to be the main factors in assessing the prognosis of PCa (21, 22). We screened the independent risk factors of PCa BM of our study to constructed clinical prediction model, which showed good prediction value (AUC = 0.833 in the testing dataset). However, there are lots of shortcomings using clinical factor to predict PCa BM. The PSA test lacks specificity: prostate hyperplasia, prostate inflammation, prostate trauma, and urinary stones can cause disruption of the prostate peritoneal barrier, which can lead to an increase in PSA (23). The results of GS are related to the site of puncture, the material used for the specimen and the subjective judgment of the pathologists, some subjective and objective errors could not be completely avoided (24).The cTx results are dependent on the co-diagnosis of pathologists and imaging physicians (25). Therefore, we should explore more objective and effective features to predict the occurrence of PCa BM.

Previously, MRI radiomics has been proved to be effective in the diagnosis and prognostic assessment of PCa patients. Numerous studies have demonstrated the value of radiomics features extracted from single or multiple scan sequences of prostate MRI in the detection and differential of PCa (26), assessment of invasiveness (27), prediction of biochemical recurrence (12)and treatment response (28). In recent years, there has been a gradual increase in the number of studies on the radiomics value on prediction of PCa BM. Zhang found that the radiomics nomogram, which incorporates the multiparametric MRI-based radiomics signature and clinical risk factors, can be used to promote individualized prediction of BM in patients with newly diagnosed PCa with the AUC of 0.92 (29).Our study came to a similar conclusion to theirs. Our study also found that T2WI and ADC intratumoral radiomic model can be used to predict PCa BM well with the AUC of 0.850.

Currently, there is few PCa-related peritumoral radiomics studies. Bai et al. found that peritumoral radiomics can better predict the presence of preoperative PCa extraperitoneal invasion compared to intratumoral radiomic and clinical features (30). Algohary et al. found that peritumoral radiomics have good predictive efficacy for PCa risk stratification (31). Our study analyzed the relationship between peritumoral MRI radiomics model and BM. In our study, the peritumoral radiomic model’s predictive efficacy was lower than that of the intratumoral radiomic model (AUC = 0.850), but it still had a relatively high predictive value for BM (AUC = 0.788), which complemented previous MRI radiomics for the prediction of PCa BM without analysis of the peritumoral region. Bova et al. found that the correlation between histological changes in the peritumoral zone and proliferative activity of tumor cells is a predictor of cancer progression (32). In addition, Algohary et al. found that a relatively higher concentration of peritumoral epithelial cells and lymphocytes were identified of high-risk lesions, which was reflected in terms of higher heterogeneity observed on T2WI peritumoral radiomic features compared to low-risk lesions (31). Thus, MRI peritumoral radiomic features may reflect different microenvironments around the tumor and the status of the tumor to some extent (33). We further merged the intratumoral with the peritumoral radiomic features for the prediction of PCa BM and obtained AUC of 0.867, which shows combined analysis of intratumoral and peritumoral information allows for a more complete portrayal of tumor heterogeneity. Peritumoral radiomic model could add additional diagnostic efficacy for single intratumoral radiomic model.

Among the five predictive models in our study, the clinic-radiomic model achieved the best efficiency, whose predictive values were better than any other models with the AUC of 0.893 in the testing dataset, suggesting that this model may promote prediction of PCa BM. The clinic-radiomic model includes not only clinical information but also radiomic features; hence in our future work, we can take a more comprehensive view to predict PCa BM, which may be more useful for clinician’s decision making and bring greater benefits to patients.

### Limitations and prospects

First, this study is a retrospective, single-institution study with a small sample size, which makes selection bias inevitable. In the future, the sample size can be further expanded and an external validation design could be adopted to verify the model efficacy. Second, the metastatic lesions detected by PET-CT or 99mTc-MDP whole-body skeletal imaging were not confirmed by pathology, so there was a certain possibility of false positives. Third, this study did not include peritumoral areas larger than 3 mm in the construction of the peritumoral model, and the performance of peritumoral areas of different ranges should be further explored in the future. Finally, the present study did not further investigate the relationship between the long-term prognosis of PCa BM patients, which can be further explored by enriching the content of the study in the future.

## Conclusion

In summary, T2WI and ADC intratumoral and peritumoral radiomic models could be used to noninvasively predict the primary diagnosis of PCa BM, and peritumoral radiomic model could add additional diagnostic efficacy. The combined clinic-imaging model can significantly improve the predictive efficacy of prostate cancer bone metastasis and can be used as an effective aid in clinical decision-making.

## Data Availability

The data analyzed in this study is subject to the following licenses/restrictions: This dataset is a retrospective collection of hospital patient data. Requests to access these datasets should be directed to Shiqian Lin, linsq6898@163.com.
