# Supplementary material for: Prediction of bone metastasis of prostate cancer based on intratumoral and peritumoral radiomics of MRI T2WI combined with ADC images
Source: Front Oncol. 2025 Mar 10;15:1555315. doi: 10.3389/fonc.2025.1555315 (PMC11930804; doi:10.3389/fonc.2025.1555315)
Supplement: Supplementary file 1 [file Table1.docx]

Table S1: MRI acquisition parameters

Table S2: Radiomics features extracted in this study

Table S3: Checklist for Artificial Intelligence in Medical Imaging

Table 1：MRI acquisition parameters

| Machine | Sequence | b value  (sec/mm^2^) | TR  (ms) | TE  (ms) | Slice Thickness  (mm) | Spacing  (mm) | FOV  (mm) | Matrix |
| --- | --- | --- | --- | --- | --- | --- | --- | --- |
| Siemens Skyra | T2WI | - | 6000 | 99 | 3.0 | 0.6 | 160×160 | 256×192 |
|  | DWI | 0,1000,2000 | 3800 | 78 | 3.0 | 0.6 | 360×220 | 100×80 |
| Siemens Magnetom Verio | T2WI | - | 5960 | 93 | 3.0 | 0.6 | 160×100 | 256×100 |
|  | DWI | 50,800,1500 | 6900 | 93 | 3.0 | 0.6 | 260×85 | 160×75 |
| Siemens Prisma | T2WI | - | 3000 | 103 | 3.0 | 0.6 | 200×200 | 384×268 |
|  | DWI | 0,1000,2000 | 4200 | 65 | 3.0 | 0.6 | 200×200 | 114×114 |
| Philips  Ingenia | T2WI | - | 3000 | 100 | 3.0 | 0.6 | 180×180 | 260×195 |
|  | DWI | 0,1000,2000 | 6000 | 86 | 3.0 | 0.6 | 240×240 | 88×84 |

Note: TR: Repetition Time; TE: Echo Time; FOV: Field of View

Table S2: Radiomics features extracted in this study

| Classification | Feature | Number |
| --- | --- | --- |
| morphological | Voxel Volume, Maximum 3D Diameter, Mesh Volume, Major Axis Length, Sphericity, Least Axis Length, Elongation, Surface Volume Ratio, Maximum 2D Diameter Slice, Flatness, Surface Area, Minor Axis Length, Maximum 2D Diameter Column, Maximum 2D Diameter Row | 14 |
| first-order | Interquartile Range, Skewness, Uniformity, Median, Energy, Robust Mean Absolute Deviation, Mean Absolute Deviation, Total Energy, Maximum, Root Mean Squared, 90 Percentile, Minimum, Entropy, Range, Variance, 10 Percentile, Kurtosis, Mean | 18 |
| GLCM | Joint Average, Joint Entropy, Cluster Shade, Maximum Probability, Idmn, Joint Energy, Contrast, Difference Entropy, Inverse Variance, Difference Variance, Idn, Idm, Correlation, Autocorrelation, Sum Entropy, Sum Squares, Cluster Prominence, Imc2, Imcl, Difference Average, Id, Cluster Tendency, Sum Average, MCC | 24 |
| GLDM | Gray Level Variance, High Gray Level Emphasis, Dependence Entropy, Dependence Non-Uniformity, Gray Level Non-Uniformity, Small Dependence Emphasis, Small Dependence High Gray Level Emphasis, Dependence Non-Uniformity Normalized, Large Dependence Emphasis, Large Dependence Low Gray Level Emphasis, Dependence Variance, Large Dependence High Gray Level Emphasis, Small Dependence Low Gray Level Emphasis, Low Gray Level Emphasis | 14 |
| GLRLM | Short Run Low Gray Level Emphasis, Gray Level Variance, Low Gray Level Run Emphasis, Gray Level Non-Uniformity Normalized, Run Variance, Gray Level Non-Uniformity, Long Run Emphasis, Short Run High Gray Level Emphasis, Run Length Non-Uniformity, Short Run Emphasis, Long Run High Gray Level Emphasis, Run Percentage, Long Run Low Gray Level Emphasis, Run Entropy, High Gray Level Run Emphasis, Run Length Non-Uniformity Normalized | 16 |
| GLSZM | Gray Level Non-Uniformity, Gray Level Non-Uniformity Normalized, Gray Level Variance, High Gray Level Zone Emphasis, Large Area Emphasis, Large Area High Gray Level Emphasis, Large Area Loy Gray Level Emphasis, Low Gray Level Zone Emphasis, Size Zone Non-Uniformity, Size Zone Non-Uniformity Normalized, Small Area Emphasis, Small Area High Gray Level Emphasis, Small Area Low Gray Level Emphasis, Zone Entropy, Zone Percentage, Zone Variance | 16 |
| NGTDM | Busyness, Coarseness, Complexity, Contrast, Strength | 5 |

Note: GLCM: Gray level co-occurrence matrix; GLDM: Gray level dependence matrix; GLRLM: Gray level run length matrix; GLSZM: Gray level size zone matrix; NGTDM: Neighborhood gray-tone difference matrix, 3D: Three demention; 2D: Two demention; Id: Inverse Difference; Idmn: Inverse Difference Moment Normalized; Idm: Inverse Difference Moment; Idn: Inverse Difference Normalized; Imc: Informal Measure of Correlation; MCC: Maximal Correlation Coefficient.

| Table 6：Checklist for Artificial Intelligence in Medical Imaging | | | |
| --- | --- | --- | --- |
| Section/Topic | No. | Item | Comply with the item or not(±) |
| TITLE or  ABSTRACT | 1 | Identification as a study of AI methodology, specifying the category of technology used (eg, deep learning) | + |
| ABSTRACT |  |  |  |
|  | 2 | Structured summary of study design, methods, results, and conclusions | + |
| INTRODUCTION |  |  |  |
|  | 3 | Scientific and clinical background, including the intended use and clinical role of the AI approach | + |
|  | 4 | Study objectives and hypotheses | + |
| METHODS |  |  |  |
| Study Design | 5 | Prospective or retrospective study | + |
|  | 6 | Study goal, such as model creation, exploratory study, feasibility study, noninferiority trial | + |
| Data sources | 7 | Data sources | + |
|  | 8 | Eligibility criteria: how, where, and when potentially eligible participants or studies were identified (eg, symptoms, results from previous tests, inclusion in registry, patient-care setting, location, dates) | + |
|  | 9 | Data preprocessing steps | + |
|  | 10 | Selection of data subsets, if applicable | + |
|  | 11 | Definitions of data elements, with references to common data elements | + |
|  | 12 | De-identification methods | + |
|  | 13 | How missing data were handled | + |
| Ground Truth | 14 | Definition of ground truth reference standard, in sufficient detail to allow replication | - |
|  | 15 | Rationale for choosing the reference standard (if alternatives exist) | + |
|  | 16 | Source of ground truth annotations; qualifications and preparation of annotators | + |
|  | 17 | Annotation tools | - |
|  | 18 | Measurement of inter-and intrarater variability; methods to mitigate variability and/or resolve discrepancies | + |
| Data Partitions | 19 | Intended sample size and how it was determined | + |
|  | 20 | How data were assigned to partitions; specify proportions | + |
|  | 21 | Level at which partitions are disjoint (eg, image, study, patient, institution) | + |
| Model | 22 | Detailed description of model, including inputs, outputs, all intermediate layers and connections | + |
|  | 23 | Software libraries, frameworks, and packages | + |
|  | 24 | Initialization of model parameters (eg, randomization, transfer learning) | + |
| Training | 25 | Details of training approach, including data augmentation, hyperparameters, number of models trained | - |
|  | 26 | Method of selecting the final model | + |
|  | 27 | Ensembling techniques, if applicable | - |
| Evaluation | 28 | Metrics of model performance | + |
|  | 29 | Statistical measures of significance and uncertainty (eg, confidence intervals) | + |
|  | 30 | Robustness or sensitivity analysis | + |
|  | 31 | Methods for explainability or interpretability (eg, saliency maps) and how they were validated | - |
|  | 32 | Validation or testing on external data | - |
| RESULTS |  |  |  |
| Data | 33 | Flow of participants or cases, using a diagram to indicate inclusion and exclusion | - |
|  | 34 | Demographic and clinical characteristics of cases in each partition | + |
| Model performance | 35 | Performance metrics for optimal model(s) on all data partitions | + |
|  | 36 | Estimates of diagnostic accuracy and their precision (such as 95% confidence intervals) | + |
|  | 37 | Failure analysis of incorrectly classified cases | - |
| DISCUSSION |  |  |  |
|  | 38 | Study limitations, including potential bias, statistical uncertainty, and generalizability | + |
|  | 39 | Implications for practice, including the intended use and/or clinical role | + |
| OTHER INFORMATION |  |  |  |
|  | 40 | Registration number and name of registry | - |
|  | 41 | Where the full study protocol can be accessed | - |
|  | 42 | Sources of funding and other support; role of funders | - |
